# Single nucleotide polymorphism and expression of genes for immune competent cell proliferation and differentiation in radiation-exposed individuals

**DOI:** 10.18699/VJ20.632

**Published:** 2020-07

**Authors:** E.A. Blinova, V.S. Nikiforov, M.A. Yanishevskaya, A.А. Akleyev

**Affiliations:** Urals Research Center for Radiation Medicine, Chelyabinsk, Russia Chelyabinsk State University, Chelyabinsk, Russia; Urals Research Center for Radiation Medicine, Chelyabinsk, Russia; Urals Research Center for Radiation Medicine, Chelyabinsk, Russia Chelyabinsk State University, Chelyabinsk, Russia; Urals Research Center for Radiation Medicine, Chelyabinsk, Russia South-Urals State Medical University of the Ministry of Healthcare of Russian Federation, Chelyabinsk, Russia

**Keywords:** exposed persons, mRNA, single-nucleotide polymorphism, real-time PCR, modification of gene expression, облученные лица, мРНК, однонуклеотидный полиморфизм, ПЦР в реальном времени, модификация экспрессии генов

## Abstract

It is known that ionizing radiation influences the expression of the genes that play a key role in the mechanisms of maintaining the stability of cellular homeostasis. As a rule, changes in the transcriptome of an exposed cell occur within the first 24 hours following radiation exposure. And it predetermines early response in the case of genome damage. Later on modulations in gene transcription activity are also possible and could result in a carcinogenic effect. However, in order to find the role of exogenous factors (ionizing radiation), it is also necessary to take into account the contribution of endogenous factors that are able to modify gene transcription activity. This is especially important for long after the onset of radiation exposure. Single nucleotide polymorphisms located in regulatory regions of the genes may belong to this group of factors. The objective of the current study was to analyze the influence of ionizing radiation on the transcription activity of the STAT3, GATA3, NFkB1, PADI4 genes, which regulate proliferation and differentiation of immune competent human cells; and to assess the potential influence of single nucleotide polymorphisms located in regulatory regions of the genes on the amount of mRNA. The study involved people who had been chronically exposed due to releases of radioactive waste into the Techa River. It was observed that 60 years after the onset of radiation exposure changes in the transcription activity of the NFkB1 and PADI4 genes were registered in people with cumulative doses to RBM within the range 78–3510 mGy. In people who had been chronically exposed, the effect of allelic variations in rs1053023, rs4143094, rs28362491, rs874881 on the level of mRNAs of the STAT3, GATA3, PADI4, NFkB1 genes has not been established.

## Abstract (ru)

Известно, что ионизирующее излучение влияет на экспрессию генов, выполняющих ключевую роль в механизмах поддержания стабильности клеточного гомеостаза. Как правило, изменение в транскрип- томе облученной клетки происходит в первые часы и сутки после радиационного воздействия, что обуслов- ливает ее ранний ответ при повреждении генома. В отдаленном периоде также возможны модуляции в транс- крипционной активности генов, приводящие к развитию канцерогенных эффектов облучения. Однако для установления роли экзогенных факторов (ионизирующего излучения) в модификации экспрессии генов кле- точного гомеостаза необходимо учитывать и роль эндогенных факторов, способных модифицировать транс- крипционную активность генов, что особенно актуально в отдаленном периоде после начала радиационного воздействия. К таким факторам могут относиться полиморфные варианты генов, расположенные в регуля- торных областях. Цель настоящего исследования – анализ влияния ионизирующего излучения в отдаленном периоде на содержание мРНК генов STAT3, GATA3, NFkB1, PADI4, регулирующих процессы пролиферации и диф- ференцировки иммунокомпетентных клеток человека, а также оценка связи аллельных вариаций rs1053023, rs4143094, rs28362491, rs874881 на количество мРНК генов STAT3, GATA3, PADI4, NFkB1. Исследование проведе- но у лиц, подвергшихся аварийному хроническому радиационному воздействию в результате сбросов радио- активных отходов в реку Течу. Установлено, что спустя 60 лет после начала радиационного воздействия у лиц, имеющих кумулятивные дозы облучения ККМ в диапазоне от 78 до 3510 мГр, регистрируются изменения в транскрипционной активности генов NFkB1 и PADI4. Не выявлено влияния аллельных вариаций rs1053023, rs4143094, rs28362491, rs874881 на количество мРНК генов STAT3, GATA3, PADI4, NFkB1 у облученных лиц.

## Introduction

Ionizing radiation induces changes in transcription activity of the genes that have a pivotal role in mechanisms of maintaining the stability of cellular homeostasis. However, the profile of gene expression differs considerably under exposure at low to high doses (Ding et al., 2005). It has been shown that exposure at low to medium doses leads to increase not only in the expression of genes involved in the response to DNA damage, but also genes of apoptosis activation (Azimian et al., 2015), cytoskeleton elements and transport of secretory vesicles (Woloschak et al., 1990), cell proliferation and differentiation (Amundson et al., 2003), as well as genes of lymphocyte activation, cytokine and chemokine expression (Wyrobek et al., 2011). It is well-known that changes in the transcriptome of an exposed cell occur within the first hours and days following the radiation exposure. It predetermines the early response in case of genome damage.

Aberrant expression of a number of genes is registered in the long-term period as well. The studies of (Fachin et al., 2009; Ilienko, Bazyka, 2016) show changes in the transcription activity of the genes, the products of which regulate intracellular transport, DNA reparation, immune response of cells 10–20 years after the onset of radiation exposure. Earlier on we have also discovered that people who have been chronically exposed at medium to high doses (0.1–4.5 Gy) as compared to unexposed individuals demonstrate a decrease in the amount of mRNA of the anti-apoptotic BCL2 gene more than 60 years after the radiation exposure (Nikiforov et al., 2019).

At the molecular level changes in the expression of genes that code various enzymes and regulatory proteins may lead to changes in the number of reactive oxygen intermediate, disbalance of the pro- and anti-inflammatory cytokines and chemokines (Barnes, Karin, 1997). However, it should be taken into account that besides the exogenous environmental factors, including ionizing radiation, the level of gene transcription activity could be influenced by endogenous (genetic) factors. In this respect, to define the role of ionizing radiation in the changes of gene transcription activity in the long-term period it is necessary to take account of genetic component.

In recent decades single nucleotide polymorphism (SNP) has been extensively studied as a marker associated with various diseases (Visscher et al., 2012; Tan, 2017). The mechanism allowing the polymorphism to influence the phenotype is determined first and foremost by the functional role of the DNA sequence where it is located. SNP can influence both the structure and activity of the gene product as well as its amount.

Of all the SNP located in the coding sequences of a gene (exons), about 58 % are non-synonymous and could affect enzymatic activity of a protein, its stability, ligand binding to a certain protein. They may lead to changes in the protein folding process and thus result in alterations of the formation of its quaternary structure (Bhattacharya et al., 2017). Other SNP are synonymous and could modify the protein expression level by influencing the secondary structure of a mature mRNA (Robert, Pelletier, 2018), promote changes of the mRNAmediated regulation of gene expression (Brest et al., 2011). Besides changing the expression level, synonymous SNP could influence mRNA stability and splicing (Wang et al., 2015).

The influence on the phenotype of SNP located in introns is largely predetermined by modifications in various regulatory elements (Shastry, 2009). Several possible mechanisms of the influence on the phenotype of SNP located in introns are known. Among these are changes in the cis-regulatory elements – enhancers and silencers modules leading to changes in trans-factors binding to these elements and to the corresponding variation of the expression level (Campbell et al., 2016), as well as other possible mechanisms of the influence of intron SNP on the level of gene expression, for example due to formation of additional chromatin loops (Wright et al., 2010).

The objective of the present study has been the analysis of the effect of ionizing radiation in the long-term period on the mRNA level of STAT3, GATA3, NFkB1, PADI4 genes that regulate the process of proliferation and differentiation of immune competent human cells, as well as the assessment of the association of alleles variation rs1053023, rs4143094, rs28362491, rs874881 with the amount of mRNA of STAT3, GATA3, PADI4, NFkB1 genes.

## Materials and methods

The study involved persons who had been chronically exposed due to releases of liquid radioactive waste of Mayak Production Association into the Techa River in 1949–1956. Residents of communities along the Techa River had combined external and internal exposure. Bottom sediments and floodplain soils contaminated with radionuclides were the sources of external γ-exposure. Internal exposure was due to radionuclide intake with river water and locally produced foodstuffs. The main dose-forming radionuclide was ^90^Sr. A β-emitter, it accumulated in bone tissue and for a long time affected the red bone marrow (RBM) (Akleyev A.V. (Ed.) The Consequences of Radioactive Pollution of the Techa River, 2016). Earlier on, increased risks of leukemia (Schüz et al., 2016) and malignant tumor (Krestinina et al., 2017) development have been proved in the cohort of exposed residents of the Techa River communities.

The study engaged 309 people with dose to RBM reconstructed with the Techa River Dosimetry System 2016 (TRDS 2016) (Degteva et al., 2019). The main group of exposed people included 163 persons with individual accumulated doses to RBM within the dose range 78–3510 mGy. For 48 persons in this group chronic exposure began during the period of their in utero development. Mean in utero dose to RBM for these people was 85 ± 12 mGy, mean postnatal dose to RBM was 506 ± 58 mGy. Another study group consisted of 115 people born before the beginning of radioactive contamination of the Techa River. Mean postnatal dose to RBM in this group was 799 ± 63 mGy. Comparison group included 146 people living in similar socio-economic conditions but with exposure dose rate to RBM not exceeding 1 mGy/year and dose accumulated over a lifetime < 70 mGy in accordance with paragraph 3.1.4 of Radiation standards 99/2009 (Sanitary Regulations and Standards SanPiN 2.6.1.2523-09). The studied group consisted of people of both sexes belonging to two ethnic groups: (1) Tartars and Bashkirs, (2) Slavs (Table 1).

**Table 1.**
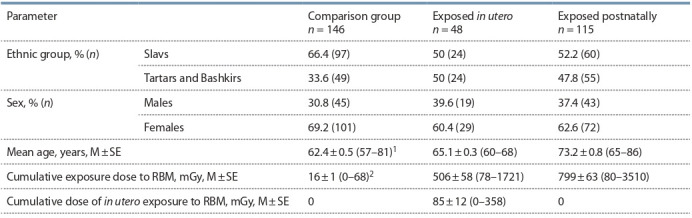
Characteristics of the studied individuals Note. RBM – red bone marrow; M – mean; SE – standard error; n – number of people; 1 – age range; 2 – range of individual dose values.

Transcription activity of genes in exposed individuals could be influenced by various factors. In this respect the following persons were excluded from the study: those who had autoimmune diseases, cancer, chronic inflammatory diseases in the exacerbation phase, those who were taking cytostatic drugs and antibiotics, those who underwent diagnostic exposure during last 6 months prior to the blood sampling, as well as those who had contact with genotoxic (chemical) agents as part of their professional activity. By the time of blood sampling all the studied individuals had undergone scheduled examination in the Urals Research Center for Radiation Medicine Clinical department within the framework of the program of healthcare provision to the exposed population (over the period 2016–2019). According to international norms in force all the examined subjects gave written informed consent to participate in the study. Research has been approved by the Institutional Review Board of the Urals Research Center for Radiation Medicine of FMBA of Russia.

Transcription activity of STAT3, GATA3, NFkB1 and PADI4 genes was studied with real-time PCR. In 2016–2019 venous blood samples from patients were collected directly into Tempus Blood RNA Collection Tubes (Applied Biosystem, USA). Native RNA was isolated immediately after stabilization or after the specimens had been stored at –80 °С.

RNA extraction has been performed with GeneJET Whole Blood RNA Purification Kit (Thermo Scientific™, USA) according to the protocol provided by the manufacturer. Information on concentration and purity of extracted RNA samples was obtained with spectrophotometer NanoDrop 2000С (Thermo Scientific, USA). A 260/280 ratio for the purified RNA extracted from all the blood samples was 2.1 ± 0.02.

Reverse transcription (RT) to synthesize complementary DNA (cDNA) was performed using High-Capacity cDNA Reverse Transcription Kit (Applied Biosystem, USA) that contains recombinant reverse transcriptase M-MLV (Moloney Murine Leukemia Virus), random hexa-nano-nucleotide primers, dNTP mixture and OT buffer. According to the manufacturer’s protocol, to synthesize cDNA we used 10 μl of total RNA.

The analysis of mRNA amount was performed using quantitative polymerase chain reaction (real-time PCR) with commercial kits TaqMan (Applied Biosystem, USA). Characteristics of primers and probes used to assess mRNA expression is given in Table 2.

**Table 2.**
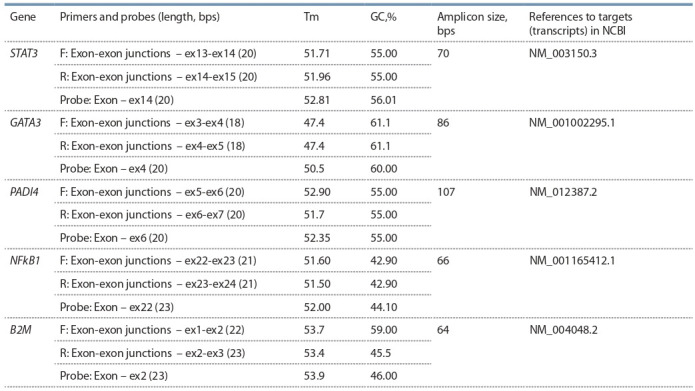
Characteristics of primers and probes to determine the content of mRNA Note. F – forward primer; R – revers primer; Tm – melting temperature; GC,% – GC content.

To obtain statistically significant results each reaction was performed three times with negative control using StepOne- Plus™ Real-Time PCR (Applied Biosystem, USA). Temperature requirements: 1 cycle of preliminary denaturation at 95 °С/10 min, then 50 cycles at 95 °С/15 s, 60 °С/1 min. The data were analyzed using the method of ΔΔCt with normalization to “housekeeping” B2M gene expression in every sample.

To be included into the study, SNP were selected based on the analysis of internet databases of genome-wide studies (www.hapmap.ncbi.nlm.nih.gov) and database of SNP (www. snpedia.com). The selection criteria were SNP location and the potential of SNP to influence gene transcription activity. Characteristics of the polymorphic regions is provided in Table 3.

**Table 3.**
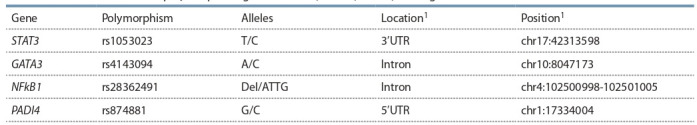
Characteristics of the polymorphic regions of STAT3, GATA3, NFkB1, PADI4 genes Note. 1 – According to whole genome database 1000Genom, version GRCh38.p12 (www.ncbi.nlm.nih.gov).

To perform genotyping we used DNA extracted from blood samples stored at –80 °C. DNA extraction from whole blood was done using the ExtraPhen kit (ATG-Biotech, Russia). Quantitative and qualitative assessment of the DNA samples after extraction was carried out with the help of spectrophotometer NanoDrop 2000C (Termo Scientific, USA).

Specimen genotyping and detection of results were performed using the real-time PCR on Applied Biosystems StepOnePlus (USA) with the reagent kit that contained primers and probes for genotyping (TestGene, Russia)

Characteristics of primers and probes for genotyping is given in Table 4.

**Table 4.**
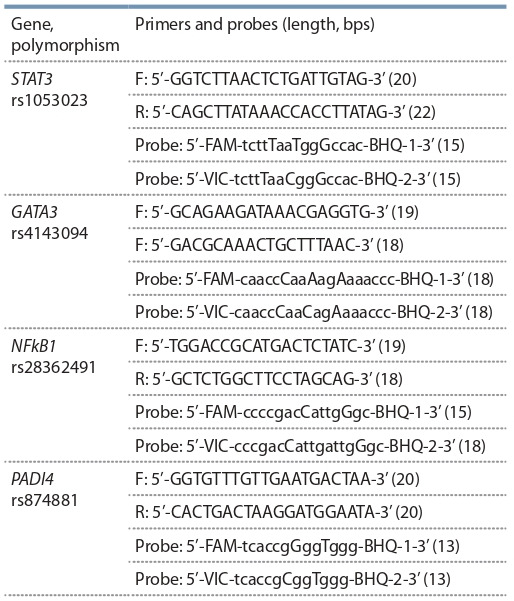
Primer and probe sequences to perform genotyping Note. F – forward primer; R – revers primer.

Amplification was carried out as directed by the manufacturer’s user’s manual guide for a particular kit. Deionized water was used as negative control.

Statistical processing of data was performed using Statistica 10.0 and WinPepi for Windows version 11.65 software packages. Kolmogorov–Smirnov’s test was used to check the normality of distribution of the mRNA amount. The obtained distribution of values differed from normal one, that is why Mann–Whitney test was applied. Genotype frequencies between ethnic groups were compared using Pearson’s χ2 test. Gene expression was assessed with р = 0.05. The significance level р = 0.01 was used in assessment of the association between SNP and gene expression.

## Results

Quantitative analysis of mRNA in all the exposed individuals testified to a decrease in the NFkB1 mRNA and increase in PADI4 mRNA relative to the comparison group. Similar patterns were registered in the group of people exposed both in utero and postnatally as well as in the group of people exposed only postnatally (Table 5).

**Table 5.**
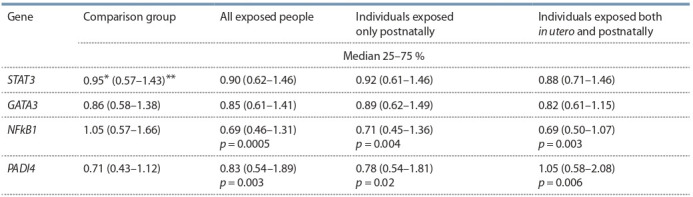
Amount of mRNA (r. u.) of the studied genes in the peripheral blood cells of chronically exposed individuals Note. Hereinafter: * – median; ** – 25 % and 75 % quartiles; p – significance level of differences in parameters between the group of exposed individuals and comparison group.

No statistically significant differences were revealed in comparative analysis of median values of mRNA of the studied genes in representatives of different ethnic groups (Tartars/ Bashkirs and Slavs) in the group of exposed individuals and in the comparison group (Table 6).

**Table 6.**
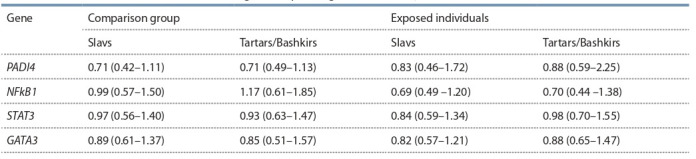
Amount of mRNA (r. u.) of the studied genes depending on the ethnicity of the studied individuals

Table 7 presents the distribution of frequencies of polymorphic loci of the studied genes in exposed people.

**Table 7.**
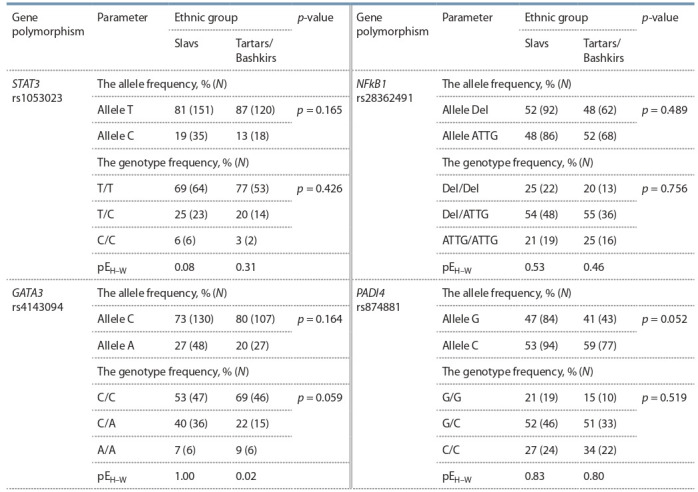
Distribution of genotypes by the studied polymorphic loci in the group of exposed individuals Note. p-value – significance of difference of allele and genotype frequency between Slavs and Tartars/Bashkirs; pEH–W – Hardy–Weinberg equilibrium.

In ethnic groups of Slavs and Tartars/Bashkirs the genotype distribution for all the polymorphic regions corresponded to the expected distribution in accordance with the Hardy– Weinberg law, except for the polymorphic region rs4143094 of GATA3 gene in the group of Tartars/Bashkirs. Moreover, distribution of alleles and genotypes did not differ between the groups of Slavs and Tartars/Bashkirs.

No association of allele variations rs1053023, rs4143094, rs28362491, rs874881 and mRNA amount of STAT3, GATA3, PADI4, NFkB1 genes was established in the group of Slavs, group of Tartars and Bashkirs and in the pooled population (Table 8).

**Table 8.**
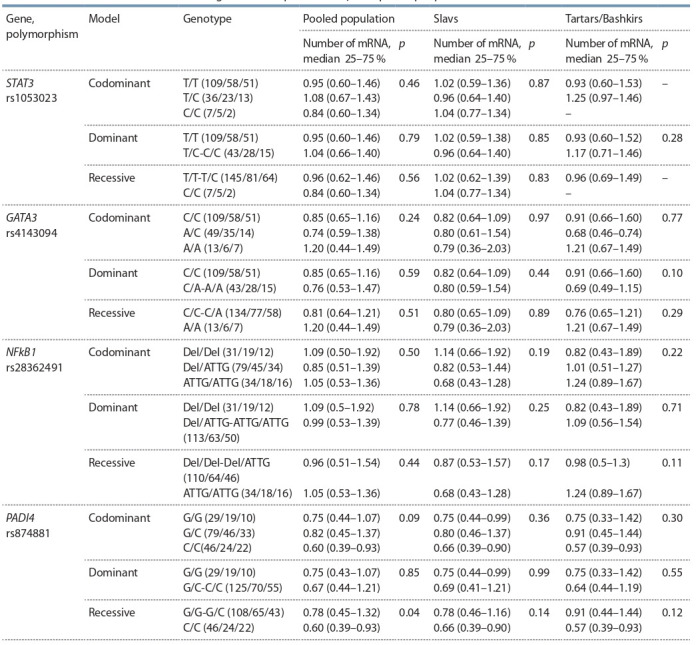
Assessment of SNP influence on gene transcription activity in exposed people

## Discussion

We have stated that 60 years after the onset of radiation exposure people with cumulative doses to RBM in the range 78–3510 mGy showed changes in the transcription activity of NFkB1 and PADI4 genes as compared to the comparison group (with doses to RBM < 70mGy). Changes in the transcription activity of genes of immune surveillance have been earlier registered in other groups of exposed individuals. For example, excessive expression of immune surveillance and apoptosis genes was observed in persons with cumulative doses of 0.1–113.35 mGy due to trans-uranium radionuclides five years after the onset of exposure (Bazyka et al., 2018). Findings of the study of the gene expression in the Chernobyl Nuclear Plant clean-up workers also demonstrate modulation of activity of more than 100 genes, including genes of cytokines and immune response, in persons with exposure doses >400 mGy 11–12 years after the onset of radiation exposure (Albanese et al., 2007).

Transcription factors are often used as candidate markers of various pathological states of immune system as their work provides plasticity of immune-competent cell population that is observed in autoimmune diseases or malignant neoplasms. For example, in different types of cancer one may observe decrease in the functional abilities of CD8+-cells, and T-cells of effector memory (TEM-cells) start to predominate phenotypically. But at the same time increase in the number of T-cells of central memory (TCM) and short-lived effector cells (TEMRA) increases the activity of anti-tumor immunity (DuPage, Bluestone, 2016).

Earlier on, we have shown the correlation relationship between expression of NFkB1 and PADI4 genes and parameters of system immunity in exposed individuals in the group of exposed residents of the Techa riverside communities. In particular, mRNA amount correlated with the absolute number of B-lymphocytes and levels of serum IgG and IgM, and amount of PADI4 mRNA correlated with the intensity of intracellular oxygen-dependent metabolism of neutrophils (Akleyev et al., 2019). Apparently, changes in the transcription activity of NFkB1 and PADI4 genes could contribute to the changes in the work of immune system.

Besides the influence of external factors, genetic component also plays certain role in transcription activity of genes. Specifically, SNP that are located in non-coding regions (enhancers, donors of splicing and acceptor sites of introns). Such SNP could influence the level of gene expression via changes in binding sites, formation of new sites, or changes in the degree of affinity of various transcription factors to certain sites of DNA binding. However, no association of allele variations rs1053023, rs4143094, rs28362491, rs874881 with the amount of mRNA of STAT3, GATA3, PADI4, NFkB1 genes in exposed individuals have been established in the current study.

## Conclusion

Thus, people who have been affected by accidental chronic exposure demonstrate decrease in the level of NFkB1 mRNA and increase in the level of PADI4 mRNA relative to the comparison group members. No influence of allele variations rs1053023, rs4143094, rs28362491, rs874881 on the level of mRNA of STAT3, GATA3, PADI4, NFkB1 genes have been registered in exposed individuals.

In view of small number of people examined by the studied polymorphic regions the findings of the research are preliminary and require further checking with greater sampling size.

## Conflict of interest

The authors declare no conflict of interest.
